# The mitochondrial ancestor of bonobos and the origin of their major haplogroups

**DOI:** 10.1371/journal.pone.0174851

**Published:** 2017-05-03

**Authors:** Hiroyuki Takemoto, Yoshi Kawamoto, Shoko Higuchi, Emiko Makinose, John A. Hart, Térese B. Hart, Tetsuya Sakamaki, Nahoko Tokuyama, Gay E. Reinartz, Patrick Guislain, Jef Dupain, Amy K. Cobden, Mbangi N. Mulavwa, Kumugo Yangozene, Serge Darroze, Céline Devos, Takeshi Furuichi

**Affiliations:** 1 Primate Research Institute, Kyoto University, Inuyama, Japan; 2 Lukuru Foundation, Projet Tshuapa-Lomami-Lualaba (TL2), Kinshasa, Democratic Republic of Congo; 3 Bonobo and Congo Biodiversity Initiative, Zoological Society of Milwaukee, Milwaukee, Wisconsin, United States of America; 4 African Wildlife Foundation, Nairobi, Kenya; 5 Department of Anthropology, Emory University, Atlanta, Georgia, United States of America; 6 Research Center for Ecology and Forestry, Ministry of high Education and Scientific Research, Mabali, Democratic Republic of Congo; 7 Consultant Biodiversity, Sustainable Use of Natural Resources, Protected Areas Management and Adaptation to Climate Change, Bangkok, Thailand; 8 Department of Behavioral Biology, University of Liège, Liège, Belgium; University of Florence, ITALY

## Abstract

We report here where the most recent common ancestor (MRCA) of bonobos (*Pan paniscus*) ranged and how they dispersed throughout their current habitat. Mitochondrial DNA (mtDNA) molecular dating to analyze the time to MRCA (TMRCA) and the major mtDNA haplogroups of wild bonobos were performed using new estimations of divergence time of bonobos from other *Pan* species to investigate the dispersal routes of bonobos over the forest area of the Congo River’s left bank. The TMRCA of bonobos was estimated to be 0.64 or 0.95 million years ago (Ma). Six major haplogroups had very old origins of 0.38 Ma or older. The reconstruction of the ancestral area revealed the mitochondrial ancestor of the bonobo populations ranged in the eastern area of the current bonobos’ habitat. The haplogroups may have been formed from either the riparian forests along the Congo River or the center of the southern Congo Basin. Fragmentation of the forest refugia during the cooler periods may have greatly affected the formation of the genetic structure of bonobo populations.

## Introduction

**Fig 1:**
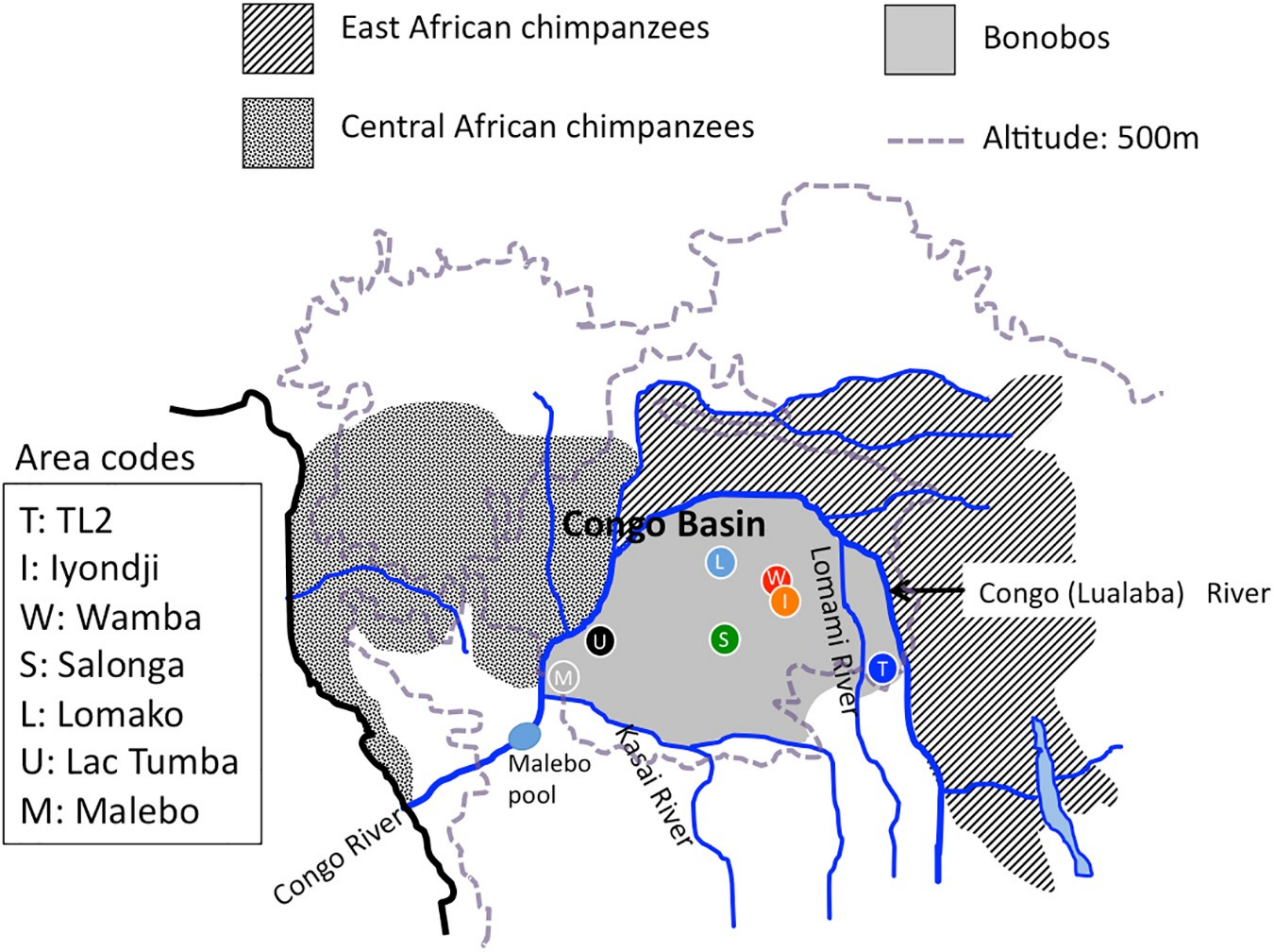
Distribution of *Pan* species in central Africa and sampling areas for mtDNA of bonobos. Sampling populations are expressed as small circles with area codes.

Bonobos (*Pan paniscus*) range in the forest area of the southern Congo Basin, the left bank of the Congo River (Fig 1). Their divergence from chimpanzees (*Pan troglodytes*) has been estimated to be 0.8–2.1 million years ago (Ma) from genetic studies [1]. Previous genome studies on two *Pan* species suggested that bonobos have clearly been separated from chimpanzees [2,3]. However the recent genome analysis indicated the possibility that some gene flow occurred between two species during the late Pleistocene [4]. In any case, we need to examine when and how the internal genetic clades of bonobos had branched off from each other, in order to investigate the history of bonobos.

Six major mtDNA haplogroups have been found in wild bonobos to date. Eriksson et al. [5] reported two haplogroups (haplogroups A and B) in five wild bonobo populations and suggested that the Lomami River acted as a riverine barrier that affected the divergence of the two mtDNA clades. Furthermore, Zsurka et al. [6] reported a third haplogroup (haplogroup C) in captive bonobos. Next, Kawamoto et al. [7] distinguished six haplogroups (A1, A2, B1, B2, C, and D) in seven wild bonobo populations. Their analysis of the relationship between the genetic distance (F_ST_) and the geographical distance among the seven populations was similar to the analysis performed by Errikson et al. [5] and indicated the linear geographical distances to show a significant correlation in the genetic distances among the populations. These results may suggest that the current riverine barrier, with the exception of the Lomami River, had a weak effect on the genetic structure of bonobos.

Kawamoto et al [7] also indicated that the east cohort (The area between the Lomami River and the Lualaba River in TL2) showed genetically closer proximity to the west cohort (Malebo and Lac Tumba) than to the central cohort (Lomako, Salonga, Wamba and Iyondji). Some haplogroups were found only within a certain cohort. For example, the A1 and C haplogroups were found in the central cohort, whereas the D haplogroup was found in the east cohort. On the other hand, the B1 haplogroup was found in two distant cohorts in the east and west, whereas the A2 and B2 haplogroups were found to be part of the two neighboring west and central cohorts. These genetic relationships may mean that the bonobo populations were not formed simply due to vicariance or dispersion. Therefore, paleoenvironment information in the Congo Basin must be reviewed, especially historical changes in the environment and the location of forest refugia during dry periods of the Pleistocene, for better understanding of the genetic structure of bonobos.

We conducted the historical analysis on the distribution of mtDNA haplogroups in bonobos. Questions to be solved were reasons for: 1) closer genetic proximity between the west and east cohort, 2) distant distribution of the B1 haplogroup, and 3) a weak effect of the current rivers on the genetic structure of bonobos. We first explored the ages and regions of the most recent common ancestor (MRCA) of bonobos and the MRCA of each haplogroup, and estimated the location of the forest refugia to be the left bank of Congo River in the Congo Basin. Then, we hypothesized the dispersal routes of bonobos after they entered the left bank of the Congo River [1].

## Methods

### DNA samples and sequencing

DNA sampling and methods for sequence determinations were previously described by Kawamoto et al. [7]. DNA samples were extracted from fecal samples, which were collected non-invasively from seven wild populations, i.e. TL2 (by HT, JH and TH), Iyondji (by TS), Wamba (by HT and NT), Salonga (by GN and PG), Lomako (by HT, JD and AC), Lac Tumba (by HT, MM and KY) and Malebo (by HT, SD and CD), that covered most of the eastern limit to the western limit of the current range of bonobos (Fig 1). We collected fecal samples from beneath of the nests or trees from which bonobos had already left, thus we never interacted or interfered with bonobos. Eighteen new fecal samples from Salonga were added to the data set of Kawamoto et al. [7] in this study.

The Scientific Authority for CITES and National Scientific Committee for the Worldwide Heritage of UNESCO in DRC has confirmed that we can publish the results obtained from the fecal samples of bonobos carried out from DRC as far as we have research permission that includes the permission to use those samples. Research Permissions during this study were issued by following authorities: 024/ICCN/BP-MA/2010 (for TL2), 051/ICCN/DG/ADG/KV/2011 (for Lomako), 1577/ICCN/ADG/ANG/DG/2008 (for Salonga) were given by the Institut Congolais pour la Conservation de la Nature (ICCN). MIN.RS/SG/002/2010 (for Iyondji), MIN.RS/SG/003/2010 (for Wamba), 008//MINRS/CREF/MAB/DG/01MNIK/2011 (for Lac Tumba) were given by Ministère de la Recherche Scientifique (MIN). 001/CREF/2012 (for Malebo) was given by the Centre de Recherche en Ecologie et Foresterie (CREF).

Obtained sequence data were deposited in DDBJ/EMBL/GenBank databases (Accession Numbers LC213801-LC213807). Sorting via gBlocks of 1117 nt sites from 154 fecal samples were identified. Haplotypes were identified by multiple alignments with ClustalX vr. 2.1 [8] of the sorted sequences.

Molecular phylogenetic relations for haplotypes were inferred using the neighbor joining (NJ) method. The mtDNA diversity within populations was estimated in terms of haplotype (gene) diversity, mean number of pairwise difference, and nucleotide diversity using the program Arlequin (v 3.5) [9]. Genetic differentiation between populations was quantified from calculations of intra- and interpopulation distances with pairwise F_ST_ distance [10] and average pairwise difference [11].

### Dating nodes of the tree

In order to estimate the time to MRCA (TMRCA) for each haplogroup, we added 44 mtDNA haplotypes of chimpanzees (*Pan troglodytes schweinfruthii*: 18, *P*. *t*. *troglodytes*: 8, *P*. *t*. *eliotti*: 7, *P*. *t*. *verus*: 11) and those of humans (9) from GenBank to the bonobos’ haplotypes (61 haplotypes). These sequence data were then sorted using gBlocks, resulting in 1073 bp aligned sequence. Each node was dated in the phylogenetic tree using the Bayesian MCMC method BEAST (2.2.0) with the HKY and Relaxed Clock Log Normal model [12,13]. The root prior was set at 6–8 Ma for Human/*Pan* divergence, and 0.95–1.05 Ma (estimation 1) and 1.7–1.8 Ma (estimation 2) for bonobo chimpanzee divergence as uniform distributions, according to the estimations from various evidences [1]. The MCMC process was performed 50,000,000 times. The phylogenetic trees generated via TreeAnnotator (v2.2) yielded the same topology for the main haplogroups that was shown by the NJ methods. These results were detected by FigTree (v. 1.4.2) (http://tree.bio.ed.ac.uk/software/figtree/).

### Evolutionary analysis

**Fig 2:**
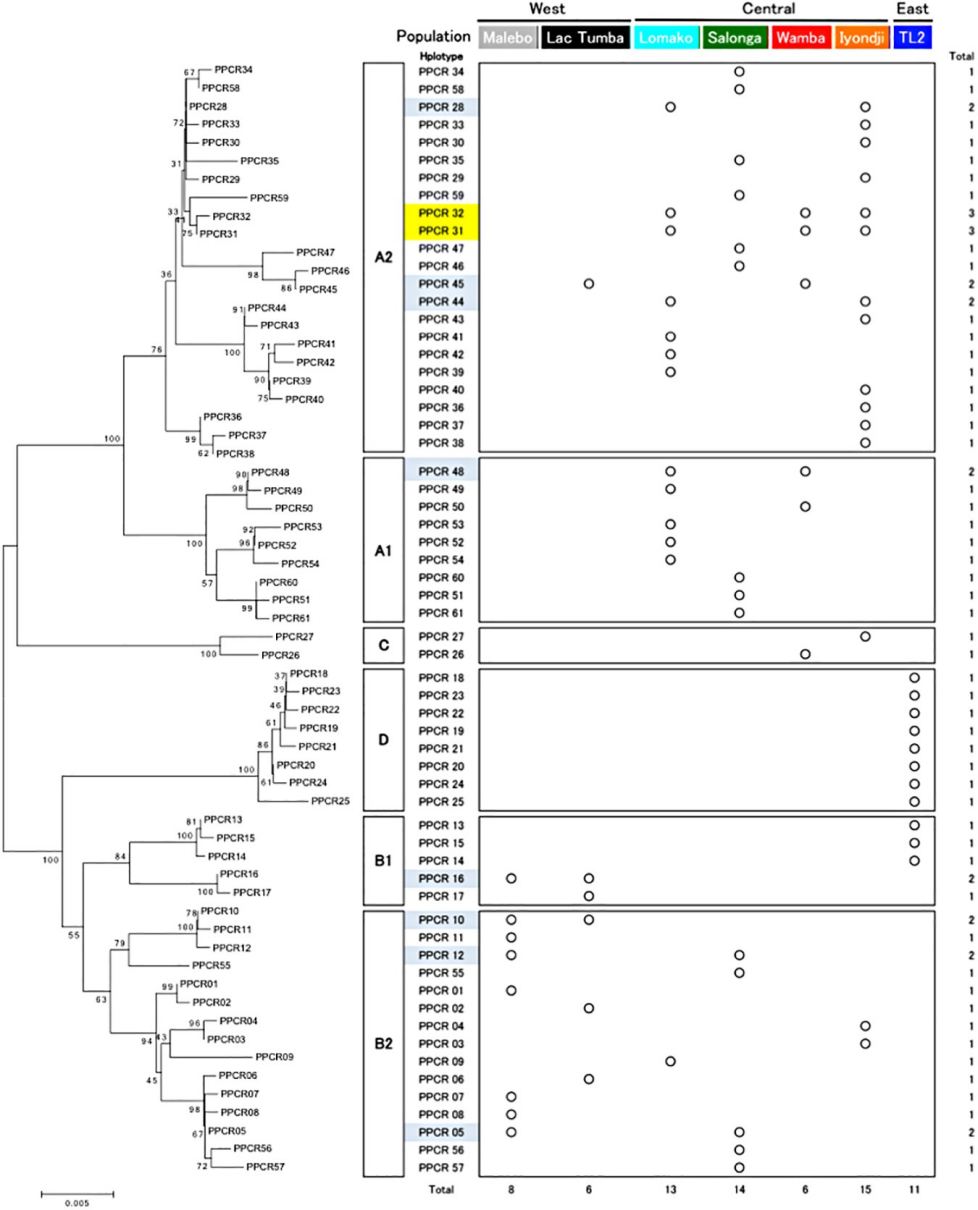
Molecular phylogeny of haplotypes and their distribution in seven populations. On the left is the phylogenetic tree constructed using the NJ method. The right side indicates the population where each haplotype was found. Blue shaded (two populations) and yellow shaded (three populations) haplotypes were found in multiple populations.

Inferring the ancestral areas for the MRCA of bonobos and evolutionary events were performed using RASP (v3.0.2) [14]. We used two different models for RASP. S-DIVA is the statistical version of DIVA (Divergence-Vicariance Analysis) [15], which is based on the parsimony of the cost-event such as dispersion or extinction. However, there is some controversy in the use of DIVA analysis for inferring population histories [16]. We limited the maximum areas for one haplotype to three areas according to the assertion of Ronquist and Sanmartín [17], because two haplotypes (PPCR31 and PPCR32) were found from three sites in the central region of A2 clade (Iyondji, Wamba, and Lomako) (Fig 2). The 50,001 MCMC trees by BEAST were used as inputs in RASP, and 50 discard trees and 500 random trees for S-DIVA were set. The Baysian Binary MCMC (BBM) method was also conducted in a similar way. The maximum area was kept to 3, with 10 chains, and 50,000 MCMC generations and JC models.

### Investigating the history of bonobos in the southern Congo Basin

First, we reviewed the reports on the paleoenvironment during the middle-to-late Pleistocene in the Congo Basin. The results of the molecular dating, the ancestral area reconstruction, and the paleoenvironment on the southern Congo Basin were combined to construct the forest areas of the southern Congo Basin during the cooler periods. From these results, we inferred how the genetic structure of bonobos was formed; namely, the history of bonobos after divergence from other *Pan* populations.

## Results

### Phylogenetic tree and haplogroup confirmation

**Fig 3:**
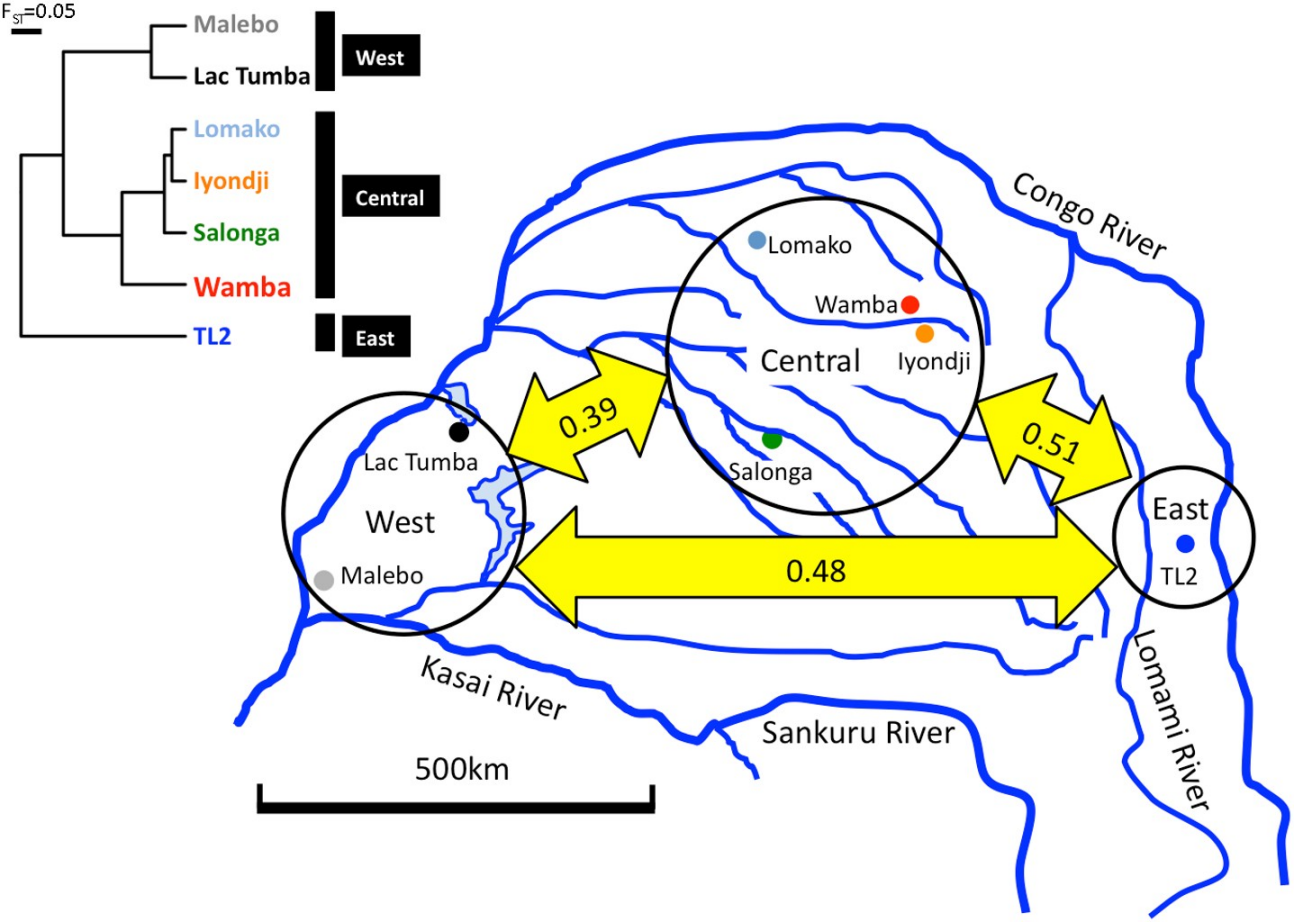
Genetic distances among three cohorts. Three circles mean east, central and west cohort respectively. Numeric values in double-headed arrows indicate F_ST_ distances among three cohorts calculated by UPGMA (the left tree).

We detected six haplogroups (A1, A2, B1, B2, C, and D) consisting of 61 mtDNA haplotypes, including 7 new haplotypes from Salonga, in the bonobo populations (Fig 2). Three cohorts (east: TL2; central: Iyondji, Wamba, Salonga, Lomako; west: Lac Tumba, Malebo) were revealed via clustering analyses and UPGMA method using F_ST_ distances (Fig 3). The F_ST_ distance among three cohorts indicated that the east cohort was genetically nearer to the west cohort than to the central cohort, which was incongruent with the geographical distances. Lac Tumba, Lomako, Salonga and Iyondji populations showed higher nucleotide diversity than the other populations (Tables 1 and 2).

**Table 1:** Genetic diversity for each cohort.

Three cohorts	West	Central	East
N	23	115	16
Nucleotide diversity	0.01540 ± 0.007931	0.020855 ± 0.010232	0.012706 ± 0.006738

**Table 2:** Genetic diversity for each population.

Seven populations	Malebo	Lac Tumba	Lomako	Salonga	Wamba	Iyondji	TL2
N	16	7	35	25	37	18	16
Nucleotide diversity	0.011588 ± 0.006173	0.022056 ± 0.012673	0.019060 ± 0.009576	0.022964 ± 0.011617	0.014601 ± 0.007396	0.018871 ± 0.009773	0.012706 ± 0.006738

### The TMRCA of bonobos and major haplogroups in bonobo populations

The TMRCA of bonobos was calculated at 0.64 Ma (0.50–0.79 with 95% HPD) by estimation 1 [0.95 Ma (0.70–1.22 Ma) by estimation 2; hereafter, a number in the square parenthesis means the age by estimation 2] (Table 3). All haplogroups diverged 0.38 Ma [0.53 Ma] or older. TMRCA of B1 haplogroup, which ranged distinctly between the west and east regions, was estimated at around 0.26 Ma [0.36 Ma]. Although a higher mutation rate of the branch was observed after divergence from chimpanzees to the MRCA of bonobos in estimation 2, there were no remarkable differences in the mutation rate among each haplogroup.

**Table 3:** Divergence time (Ma) for major clades calculated by BEAST (with 95% HPD).

Root prior (chimpanzee/bonobo)	Node 1 (TMRCA)	Node 2 (C/A)	Node 3 (B/D)	Node 4 (A1/A2)	Node 5 (B1/B2)
0.95–1.05 (Estimation 1)	0.64 (0.50–0.79)	0.57 (0.42–0.74)	0.47 (0.33–0.64)	0.44 (0.28–0.60)	0.38 (0.26–0.53)
1.7–1.8 (Estimation 2)	0.95 (0.70–1.22)	0.84 (0.62–1.19)	0.67 (0.45–0.92)	0.60 (0.38–0.85)	0.53 (0.34–0.75)

### Ancestral areas and dispersal routes

**Fig 4:**
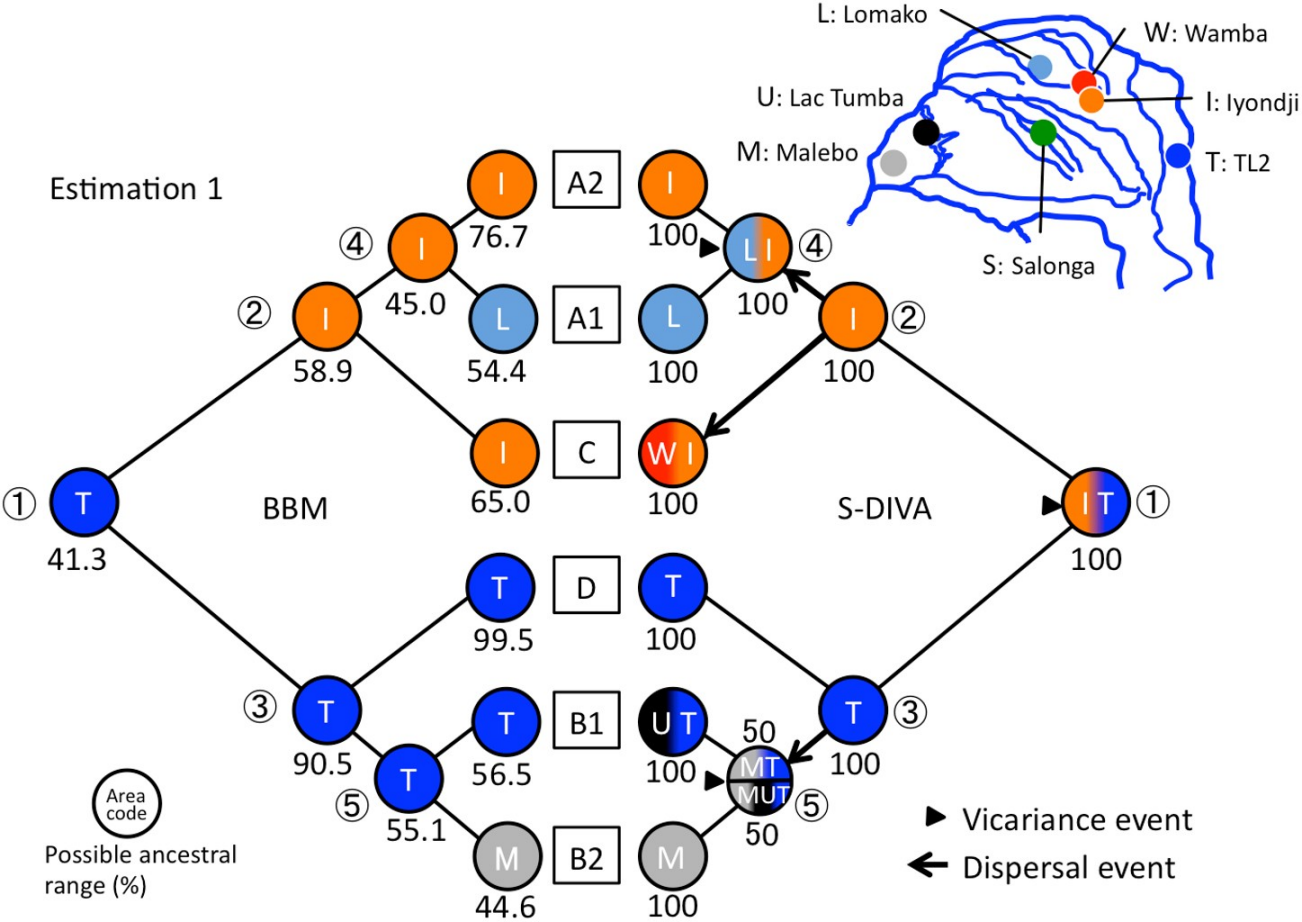
Ancestral area reconstructions by RASP based on estimation 1. Each pie graph shows the possible ancestral range (%) in each node. The same color indicates the same area. If the ancestral area of a node is indicated by more than one area, the gradient colors for these areas are used. Only the most likely state (i.e. the highest probability area) is shown in BBM. The triangles or arrows at each node show vicariance or dispersal events indicated by S-DIVA. Numbers in circles mean major nodes in the phylogenetic tree (Table 3).

**Fig 5:**
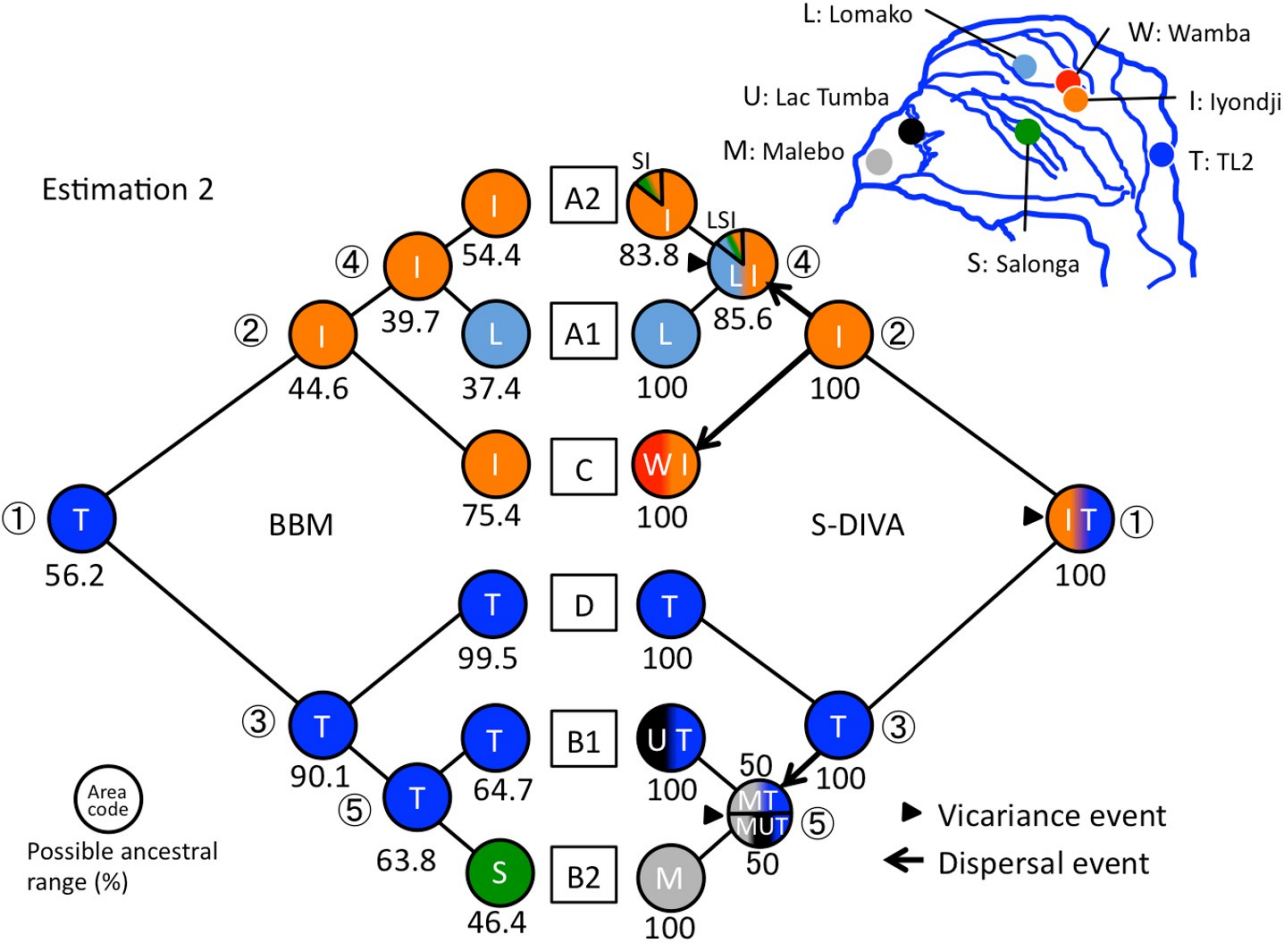
Ancestral area reconstructions by RASP based on estimation 2. Each pie graph shows the possible ancestral range (%) in each node. The same color indicates the same area. If the ancestral area of a node is indicated by more than one area, the gradient colors for these areas are used. Only the most likely state (i.e. the highest probability area) is shown in BBM. The triangles or arrows at each node show vicariance or dispersal events indicated by S-DIVA. Numbers in circles mean major nodes in the phylogenetic tree (Table 3).

Ancestral area reconstructions using the two estimations portrayed similar results (Figs 4 and 5 and Table 4). In the S-DIVA model, the ancestral range for the MRCA of the collected mtDNA samples was indicated over the TL2 and Iyondji areas, and it was separated into those two areas by a vicariance event. The A1, A2, and C haplogroups originated in the Iyondji area. The A1 and A2 haplogroups differentiated in the central region such as Iyondji or Lomako. On the other hand, the ancestral area for the B and D haplogroups was TL2. The D haplogroup was formed within TL2. The B haplogroups enlarged their range to the Malebo or Lac Tumba areas. The B1 haplogroup ranged over the TL2 and western region, and separated into east and west regions later. The ancestral range of the B2 haplogroup was estimated to be Malebo. In the BBM model, four of five major nodes indicated the plural possibility of ancestral area in both estimations (Table 4). However, the area indicated by S-DIVA showed the highest probability in most cases (Figs 4 and 5).

**Table 4:** Possible ancestral ranges (%) for each node in the phylogenetic tree indicated by RASP.

S-DIVA	Node 1 (MRCA)	Node 2 (C/A)	Node 3 (B/D)	Node 4 (A1/A2)	Node 5 (B1/B2)
Estimation 1 0.95–1.05	TL2·Iyondji (100.0)	Iyondji (100.0)	TL2 (100.0)	Iyondji·Lomako (100.0)	TL2·Malebo (50.0), TL2·Lac Tumba·Malebo (50.0)
Estimation 2 1.7–1.8	TL2·Iyondji (100.0)	Iyondji (100.0)	TL2 (100.0)	Iyondji·Lomako (85.6), Iyondji·Salonga·Lomako (14.4)	TL2·Malebo (50.0), TL2·Lac Tumba·Malebo (50.0)
BBM	Node 1 (MRCA)	Node 2 (C/A)	Node 3 (B/D)	Node 4 (A1/A2)	Node 5 (B1/B2)
Estimation 1 0.95–1.05	TL2 (41.3), Iyondji (20.0), Wamba (12.2)	Iyondji (58.9), Wamba (20.2), Lomako (7.2)	TL2 (90.5)	Iyondji (45.0), Lomako (26.1), Wamba (10.8)	TL2 (55.1), Malebo (16.9), Lac Tumba (10.1), Salonga (5.7)
Estimation 2 1.7–1.8	TL2 (56.2), Iyondji (13.3), Wamba (11.9)	Iyondji (44.6), Wamba (31.3), TL2 (8.7)	TL2 (90.1)	Iyondji (39.7), Wamba (27.8), Salonga (12.2), Lomako (7.2)	TL2 (63.8), Malebo (14.5), Lac Tumba (6.9)

Some haplotypes of B2 ranged the central region, but the central region was not indicated as a main dispersal route for the B2 haplogroup except for BBM in estimation 2. Similarly, although one haplotype (PPRC45) of A2 ranged in Lac Tumba (western region), this haplotype was concluded to be the result of recent gene flow from Salonga in the central region.

## Discussion

### Inferring the history of bonobos in the southern Congo Basin

Although previous genome studies suggested no gene flow between bonobos and any of the chimpanzee groups [2,3], some gene flow from chimpanzees to bonobos might have occurred after 0.65 Ma [4]. However mtDNA haplotypes of bonobos analyzed in this study coalesced to form one root (Figs 2, 4 and 5) and clearly separated from the haplotypes of chimpanzees. Therefore mtDNA haplotypes in this study can be thought to have originated within bonobo populations without gene flow from other *Pan* populations.

Molecular dating and reconstructing the ancestral areas for each node suggested that the MRCA of bonobos ranged the eastern areas, including TL2 and/or Iyondji, at around 0.64 Ma [0.95 Ma]. The A1, A2, and C haplogroups diverged in the central region, and the common ancestor of the B and D haplogroups originated in the eastern region TL2. The B clades appeared to disperse from TL2 to the western region but not through the central region. Therefore, we need to consider how the B clades dispersed from the eastern to western regions directly.

#### Forest refugia in the southern Congo Basin

Palynological records since 1.35 Ma from the Ocean Drilling Program (ODP: 1985–2002) 1075 site in the deep sea fan sediments of the Congo River have shown that the most prominent change in pollen assemblages occurred at 1.05 Ma [18]. An increase in *Podocarpus* pollen, coupled with decreases in tropical, woodland, and mangrove tree pollen, suggests a shift to a cooler phase in the Congo Basin. The swampy open land probably dominated in the basin according to the retrogression of the tropical forest area in the basin during LGM [19,20,21]. Therefore, the wetland represented by Cyperaceae, *Xyris*, *Nymphaea lotus*, and *Laurembergia tetrandra*, as well as the savanna, might have also expanded to other cooler stages before LGM.

Two different areas have been proposed as the forest refugia for LGM. Most of them were figured as riparian forests along the main stream of the Congo River or the water-rich area around Lac Tumaba and Mai-Ndombe [22, 23, 24, 25, 26, 27]. The others were presumed as fragment forests in the center of the southern basin and Lomami (TL2) areas [28,29]. Each refugium possibly separated into more small forest fragments during severe dry phases or connected each other during relatively warmer dry phases, but main forest areas during the cooler periods would have remained situated along the Congo River and the center of the southern Congo Basin.

**Fig 6:**
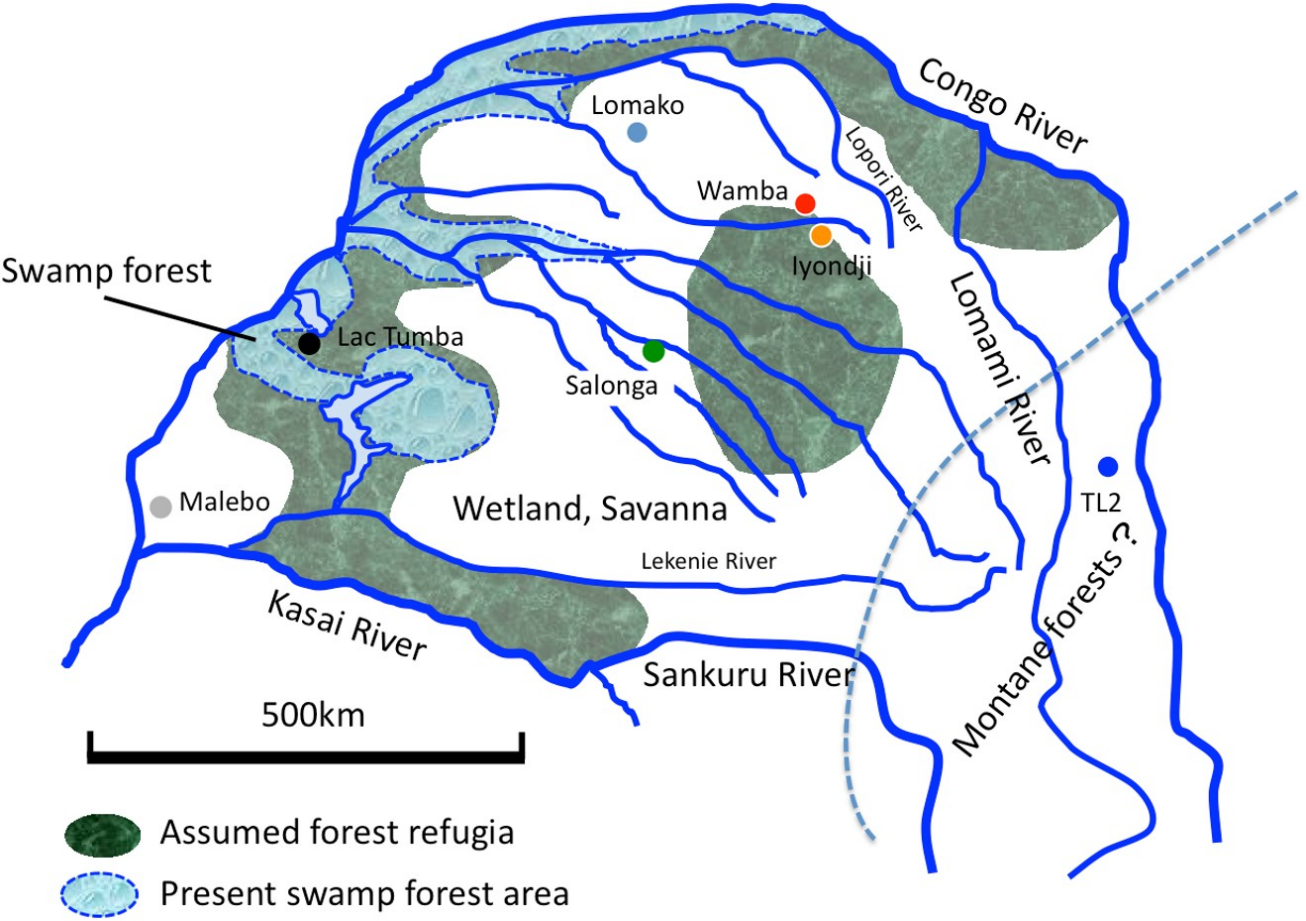
Assumed forest refugia of the bonobos’ habitat during glacial stages. Forest refugia might have existed stably along the main stream of the Congo River and the center of the southern Congo basin through cooler periods during the Pleistocene. Montane forests may have expanded to the basin in such periods (dotted line).

If both riparian forests and forests in the center of the southern Congo Basin have existed stably throughout the middle to late Pleistocene to a certain extent, the divergence through those forest refugia may explain the ancestral areas and direction of dispersion of the mtDNA haplotypes (Fig 6). The population inhabited around TL2 and Iyondji seems to have survived since 0.64 [0.95] Ma. The MRCA of haplogroups B and D seemed to have been formed around TL2 or over TL2 and the western region 0.47 Ma [0.67 Ma]. The MRCA of haplogroups A and C was born around 0.57 [0.84] Ma in the central region. Mutual exchanges of bonobo populations between these two forest refugia seem to have little possibility during the cooler stages. Montane forests, represented by *Podocarpus*, might approach from the Western Rift to the east to south regions of the bonobos’ habitat during the cooler stages in the middle Pleistocene [30].

The existence of riparian forest refugia might have been easily imagined because the area could be regarded as the water rich area. However, what maintained the forest refugia in the center of the southern Congo Basin during cooler periods remains unknown. One possibility is the higher level of groundwater level at that location. The area where the top of the basement is less deep than the other areas lies in the region that is south-west to the center of the southern Congo Basin [31,32]. This subsurface structure, called Lokonia (or Lonkonia) High, might have supported the existence of the forests during the dry phases with higher level of groundwater.

#### The dispersal routes of the bonobo populations in the left bank of the Congo River

**Fig 7:**
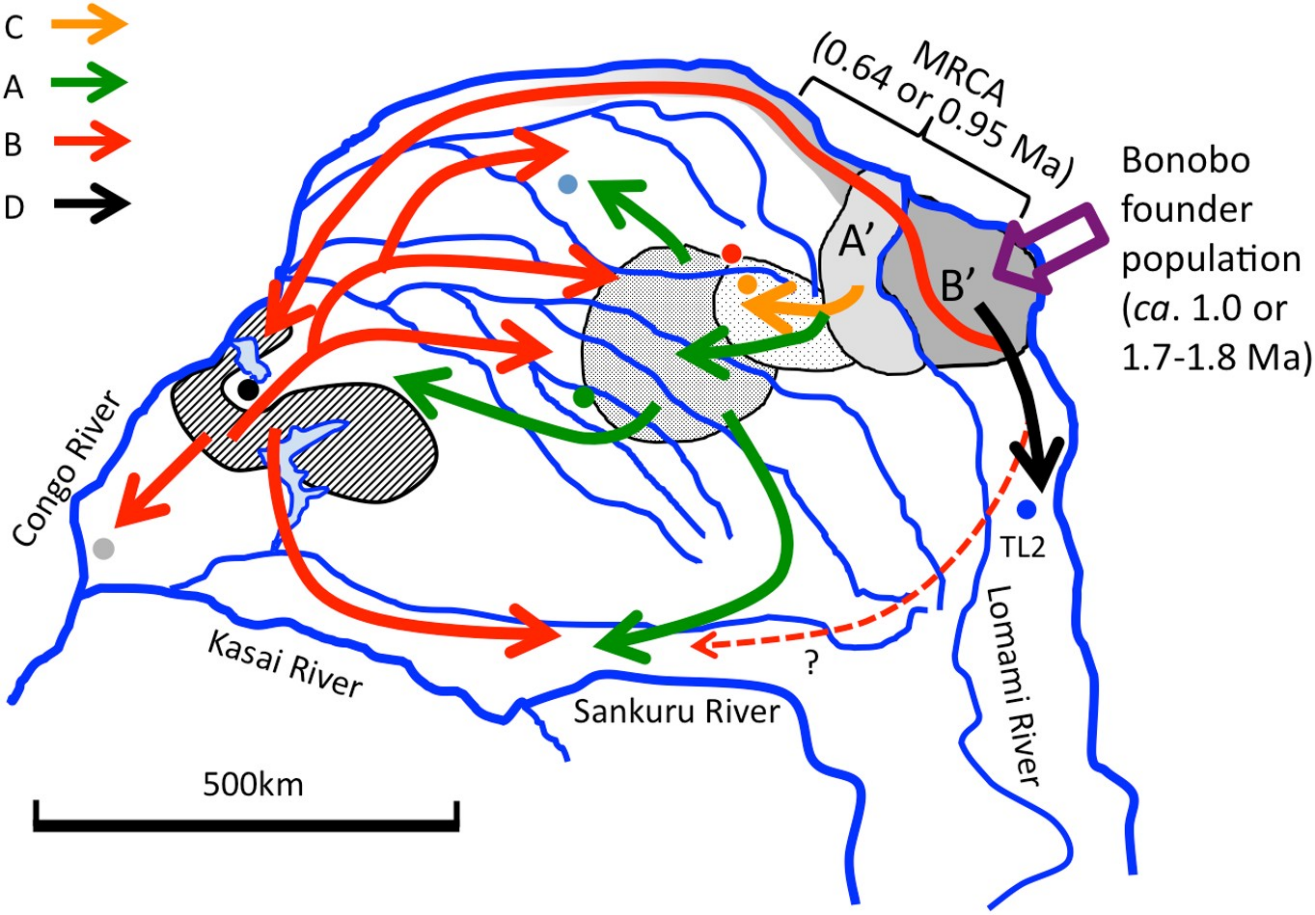
The hypothetical dispersal routs of bonobo populations since the mid-Pleistocene. The areas of A’ and B’ represent the area of the MRCA of bonobos. Other hatched areas indicate the core refugia for the main haplogroups. Arrows in each color indicates the dispersal routs of each haplogroup. A dotted red arrow means another possible dispersion route for haplogroup B.

Takemoto et al. [1] hypothesized that the bonobos’ ancestor crossed the Congo River to its left bank ca. 1.0 Ma [1.7–1.8 Ma], and that the most probable crossing point was the upper stream of the Congo River in the eastern region. The present study showed that the mtDNA ancestral populations most likely inhabited the eastern region of the current bonobos’ habitat at around 0.64 Ma [0.95 Ma] (Fig 7), supporting the previous supposition. Either 0.64 or 0.95 Ma was one of the coldest ages from 1.3 Ma [1,18, 33,34] and the bonobos’ range might have been very limited along the river. Bonobos might have crossed the Lomami River from the Lomami area to the Iyondji area during this cold age, or they might have already ranged over both banks of the Lomami River before this age. During the warm period immediately following it, the water levels of the Lomami River might have risen and divided the bonobo population across both banks of the Lomami River (A’ and B’).

The A’ population might have expanded their range to the center of the southern basin according to the increase of forest area. They would have survived in the refugia of the center of the southern basin and differentiating into the C, A1, and A2 haplogroups later. The C haplogroup remained near to the east areas such as the Iyondji and Wamba. The A2 haplogroup expanded to a wide range including the Lac Tumba of the western region, whereas the range of A1 remained in the central region. A part of the B’ population might have migrated to the left bank of the Lomami River at another age when the water level of the Lomami River decreased. They seemed to have arrived at the Malebo through the forest refugium along the Congo River to become the common ancestor of the B2 and B1 haplogroups. The B2 haplogroup enlarged its range over the western and central regions. Furthermore, the B1 haplogroup was separated into the eastern and western region at around 0.26 Ma [0.36 Ma].

Mutual exchange of populations between the western and central regions might have occurred at plural times according to the forest expansion and contraction by glacial-inter glacial cycles. The eastern region, TL2, however did not seem to be connected to the central region directly, but through the western region due to the riverine barrier of the Lomami River during the warm period and the distinct distribution of the forest refugia in the cooler periods. There is another possibility that the B clade dispersed to the western region through the southern route when the Montane forest approached near the basin. This dispersion route would have yielded the same results with the riparian forest refugia, allowing the TL2 population to migrate to the central region via the western region (Fig 7).

The eastern region was indicated as the first colonization areas in the left bank of the Congo River not only for bonobos but also for other forest living animals [1,35]. Furthermore, the morphological study of cranial measurements indicated the TL2 population was divergent slightly more from the Lukenie population, ranging between the Lukenie and Sankuru River, than from the central region [36]. This is consistent with our hypothesis because the Lukenie area is the farthest area from TL2 when the forest refugium is followed along the mainstream. The hypothesis presented here will be verified by genetic studies for the populations along the mainstream of the Congo River. This area was assumed to represent migration routes for bonobo populations. If the B1 or D haplogroup, which is restricted to the west or east cohort, are found in these areas, our hypothesis may become more plausible.

### Differences in genetic diversity among populations

The BBM analysis of estimation 2 suggested the possibility that the ancestral area of B2 was the Salonga in the central region (Fig 5). This might be caused by a geographical proximity between the Salonga and western core refugia (Fig 7). The western core refugia was probably situated between Lac Tumba and Salonga, which is now occupied by swamp forests (Fig 6). The Salonga and Lac Tumba populations showed the highest and second highest nucleotide diversities (Table 1). That may be because they are the nearest populations belonging to different cohorts that maintained the different haplogroups, A and B. In other words, the sympatric existence of A and B haplogroups in a population appears to be a causal factor for the higher nucleotide diversity in the Salonga, Iyondji, Lomako, and Lac Tumba populations.

The genetic diversity of populations usually declines from the center of a geographical range to the periphery, and the differences in the genetic diversity among the study populations matched this pattern [7]. However, the mechanism that generates this pattern is not clear [37]. The present analysis showed the possibility that the central area has a higher genetic diversity involving various genetic clades because chances of exchanges between different genetic clades with neighboring areas might be greater in the central area than in the peripheral area. It may be helpful to investigate the genetic clades in populations from the viewpoint of the historical biogeography in order to clarify the geographical pattern of the genetic diversity in these populations.

### Molecular dating for TMRCA of bonobo populations

Two studies [4, 38] estimated that the TMRCA of bonobos was 0.4–0.5 Ma and the divergence time of bonobos from chimpanzees to be around 1.8 Ma. Other four studies also suggested that TMRCA of bonobos by mtDNA was at around 0.5 Ma [4,5,6] (Table 5). If these estimations are true, three quarters or two-thirds of the bonobos’ genetic history remains unknown. The current study estimated that the TMRCA of bonobos was 0.95 (0.70–1.22) Ma, using a divergence time of 1.7–1.8 Ma for bonobos/chimpanzees and 6–8 Ma for human/*Pan*. Similarly, when using a divergence time of 0.95–1.05 Ma, TMRCA of bonobos was estimated as 0.64 Ma. Our estimations suggest that the current population of bonobos had an older origin than previously reported after the branching off from chimpanzees. Our analysis shortened the gap between the divergence time of chimpanzees/bonobos and TMRCA of bonobos.

**Table 5:** Molecular dating for TMRCA of bonobos.

	Bonobos/Chimpanzees Divergence time	TMRCA of bonobos	Calibrations and materials	Citation
Previous studies	1.8 ± 0.3 Ma	0.5 ± 0.27 Ma	Human/*Pan*: 5–7 Ma, Y chromosome	Stone et al. 2002
	-	0.54 (0.43–0.66) Ma	Human/*Pan*: 6–8 Ma, mtDNA, captive bonobos	Zsurka et al. 2010
	-	0.41–0.46 Ma	mtDNA, HV1	Eriksson et al. 2006
	-	0.04–0.045 Ma	Y chromosome	Eriksson et al. 2006
	1.74 (1.54–1.96) Ma	0.40 (0.35–0.45) Ma	Whole genome	Manuel et al, 2016
This study	(1.03 Ma)	0.64 (0.50–0.79) Ma	Human/*Pan*: 6–8 Ma, Bonobo/Chimpanzee: 0.95–1.05 Ma, mtDNA	Estimation 1
	(1.76 Ma)	0.95 (0.70–1.22) Ma	Human/*Pan*: 6–8 Ma, Bonobo/Chimpanzee: 1.7–1.8 Ma, mtDNA	Estimation 2

We delineated the history of bonobos in the southern Congo Basin from the limited DNA samples and information of the basin’s paleoenvironment. Therefore, the hypothetical history of the bonobos presented here should be renewed in the near future. At this stage, however, it is difficult to obtain DNA samples from other sites, as well as additional detailed paleoenvironmental data on the early to middle Pleistocene in the basin. Addition of mtDNA samples from new sites to our analysis or analyzing nuclear DNA may elucidate the bonobos’ population history in more detail.
